# The External Exposome and Allergies: From the Perspective of the Epithelial Barrier Hypothesis

**DOI:** 10.3389/falgy.2022.887672

**Published:** 2022-07-08

**Authors:** Zeynep Celebi Sozener, Ümüs Özbey Yücel, Seda Altiner, Betül Ozdel Oztürk, Pamir Cerci, Murat Türk, Begüm Gorgülü Akin, Mübeccel Akdis, Insu Yilmaz, Cevdet Ozdemir, Dilsad Mungan, Cezmi A. Akdis

**Affiliations:** ^1^Clinic of Immunology and Allergic Diseases, Ankara City Hospital, Ankara, Turkey; ^2^Department of Nutrition and Diet, Ankara University, Ankara, Turkey; ^3^Division of Immunology and Allergic Diseases, Department of Internal Medicine, School of Medicine, Ankara University, Ankara, Turkey; ^4^Division of Immunology and Allergic Diseases, Department of Chest Diseases, School of Medicine, Ankara University, Ankara, Turkey; ^5^Clinic of Immunology and Allergic Diseases, Eskisehir City Hospital, Eskisehir, Turkey; ^6^Clinic of Immunology and Allergic Diseases, Kayseri City Hospital, Kayseri, Turkey; ^7^Swiss Institute of Allergy and Asthma Research (SIAF), University of Zurich, Davos, Switzerland; ^8^Division of Immunology and Allergic Diseases, Department of Chest Diseases, Erciyes University, Kayseri, Turkey; ^9^Department of Pediatric Basic Sciences, Institute of Child Health, Istanbul University, Istanbul, Turkey; ^10^Division of Pediatric Allergy and Immunology, Department of Pediatrics, Istanbul Faculty of Medicine, Istanbul University, Istanbul, Turkey; ^11^Christine Kühne-Center for Allergy Research and Education (CK-CARE), Davos, Switzerland

**Keywords:** air pollution, climate change, epithelial barrier, microbiome, nutrition, exposome

## Abstract

**Introduction:**

In the last decades, we have seen a rapid increase in the prevalence of allergic diseases such as asthma, allergic rhinitis, atopic dermatitis, and food allergies. The environmental changes caused by industrialization, urbanization and modernization, including dramatic increases in air pollutants such as particulate matter (PM), diesel exhaust, nitrogen dioxide (NO2), ozone (O3), alarming effects of global warming, change and loss of biodiversity, affect both human health and the entire ecosystem.

**Objective:**

In this review, we aimed to discuss the effects of the external exposome on epithelial barriers and its relationship with the development of allergic diseases by considering the changes in all stakeholders of the outer exposome together, in the light of the recently proposed epithelial barrier hypothesis.

**Method:**

To reach current, prominent, and comprehensive studies on the subject, PubMed databases were searched. We included the more resounding articles with reliable and strong results.

**Results:**

Exposure to altered environmental factors such as increased pollution, microplastics, nanoparticles, tobacco smoke, food emulsifiers, detergents, and household cleaners, and climate change, loss and change in microbial biodiversity, modifications in the consumption of dietary fatty acids, the use of emulsifiers, preservatives and the decrease in the antioxidant content of the widely consumed western diet may disrupt the epithelial barriers of the skin, respiratory and gastrointestinal tracts, making us more vulnerable to exogeneous allergens and microbes. Epithelial cell activation, microbial dysbiosis and bacterial translocation disrupt the immune balance and a chronic Th2 inflammation ensues.

**Conclusion:**

Dramatic increases in air pollution, worrisome effects of global warming, dysbiosis, changing dietary habits and the complex interactions of all these factors affect the epithelial barriers and local and systemic inflammation. We want to draw attention to the emerging health effects of environmental changes and to motivate the public to influence government policies for the well-being of humans and the nature of the earth and the well-being of future generations.

## Introduction

The concept of the exposome covers all environmental exposures throughout an individual's life span (1, 2). These exposures can be sub-grouped into three categories: general external environment (climate, biodiversity, urban environment, social and economic factors), specific external environment (allergens, microbes, diet, tobacco, pollutants, and toxic substances), and host-dependent internal environment (metabolic factors, inflammation, and oxidative stress) (1, 3). The bidirectional effect of the environment on human beings and the effect of humans on all other living systems and genomes has been recently referred to as the meta-exposome (4).

A high biodiversity has been proposed to enrich the human microbiome, maintain immune homeostasis, and protect against allergic and inflammatory diseases (5). After the industrial revolution in the 19th century, air pollutants increased significantly in all areas with industrial sectors, especially in urban areas. These contribute to global warming, and loss of biodiversity not only in human microbiota but also in the ecosystem (3, 6, 7). Westernized (modern urban life) diets are characterized by low antioxidants and fiber intake and high fatty acid content. In addition, processed foods with additives, such as preservatives, enzymes, and emulsifiers have become an important part of the diet (8, 9). The “*epithelial barrier hypothesis”* has been recently proposed to explain the detrimental effect of all these factors on human health, attributing it to the damage of these substances to the epithelial barriers of the skin, respiratory and gastrointestinal tracts and reshaping of the microbiome, leading to initiation of peri epithelial inflammation (8).

**Figure 1:**
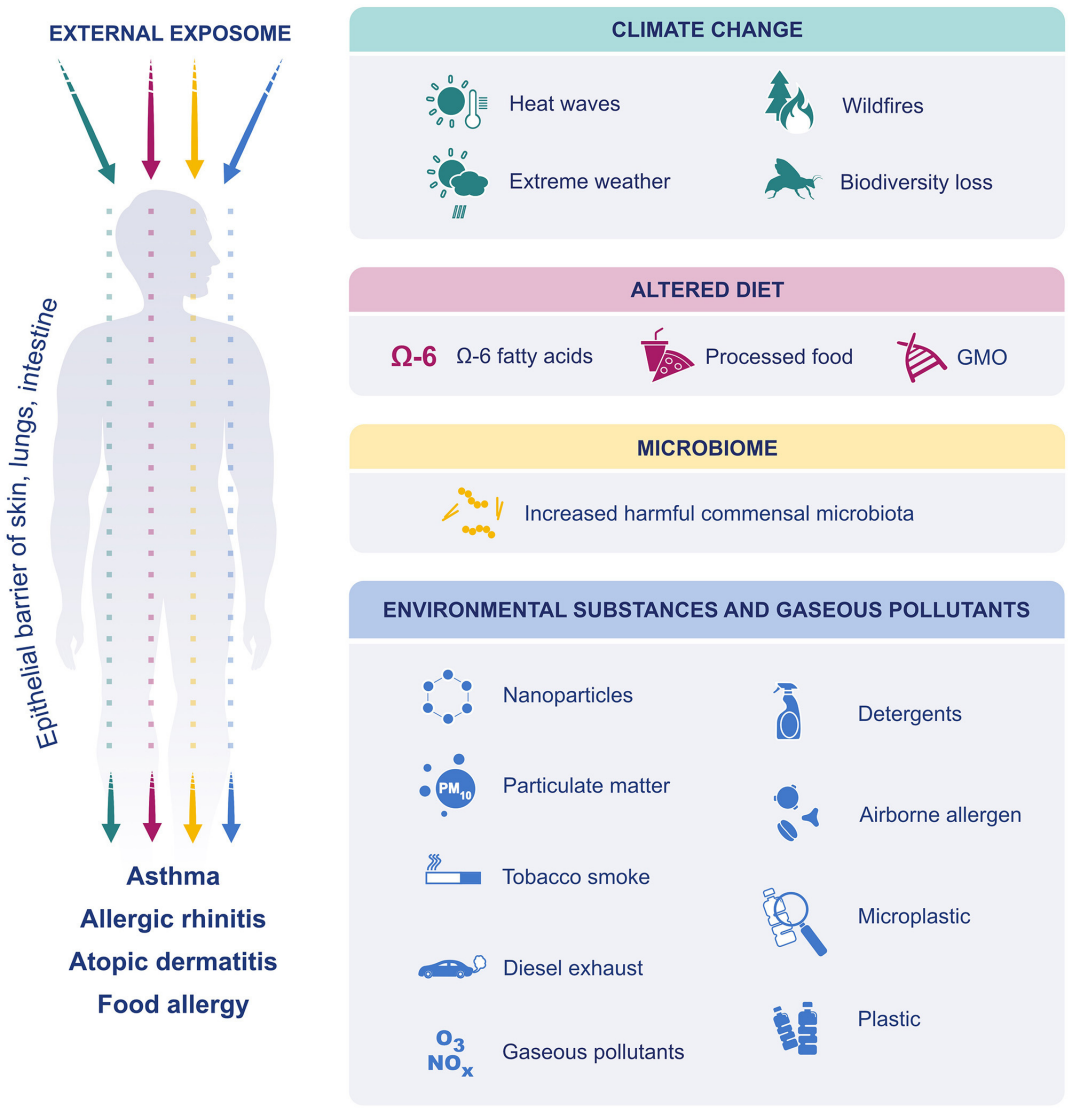
The effect of external exposome on epithelial barriers of skin lung and intestine. Due to climate change, extreme weather events have become more frequent and more intense. The air was polluted, and biodiversity was lost. Dietary preferences have shifted toward increased consumption of processed foods, n-6 fatty acids and GM foods. Exposure to environmental substances such as, detergents, PM, ozone, diesel exhaust, nanoparticles, microplastics, environmental tobacco smoke and airborne allergens were increased. Finally, the microbiome was affected, and increase in harmful commensals resulted in dysbiosis. All these factors affect and disrupt the epithelial barriers of skin, lung and gastrointestinal system and cause allergic diseases.

In this review, we discuss how these environmental and lifestyle changes including climate change, microbial dysbiosis, altered diet preferences, processed food additives, and increased exposure to environmental insults and pollutants (detergents, airborne allergens, particulate matter (PM), ozone, microplastics, nanoparticles, and tobacco smoke) affect the development of allergic diseases in relation with the recently proposed “*epithelial barrier hypotheses”* (Figure 1).

## Climate Change

Anthropogenic activities are increasing the emissions of carbon dioxide (CO_2_), methane (CH_4_), nitrogen oxides (NOx), fluorinated gases and PM in the atmosphere and cause global warming by trapping heat on Earth, the greenhouse effect, which is the biggest threat to our planet including all ecosystems (9). As a result, natural disasters are becoming more frequent and severe as observed in recent years with record numbers of heat waves, wildfires, thunderstorms, sandstorms, droughts, floods, blizzards, and hurricanes (10, 11). A warmer and more humid environment changes the cycle and seasonal nature of plants in some regions, prolongs the pollen season, increases the quantity of pollen, and alters the distribution, timing, dispersion of some pollen species (i.e., ragweed in Europe), which may lead to increase in the prevalence and severity of allergies (3, 9, 11). In addition, rising levels of CO_2_ and other air pollutants may increase pollen outputs and allergenicity (3, 12–14). Besides increased urbanization, global warming has created warm and humid conditions, which provide a suitable environment for the growth of both indoor allergens and outdoor molds (15, 16). The protease activities of house dust mites and Aspergillus fumigatus have been shown to damage the epithelial barriers (17). There are many unknowns on the impact of climate change on human health and further research is warranted to elucidate the causative relationship between climate change and different types of allergies or other inflammatory conditions. For instance, it has been shown that pollen, independent of allergens, alters innate immunity and especially impairs antiviral defense by changing the immunological barrier functions of airway epithelial cells, interfering with NF-κB signaling in dendritic cells, reducing interferon-λ and proinflammatory chemokine responses (22, 23).

The prevalence of peanut and tree nut allergies has increased during the last few decades, but there is insufficient evidence to link this phenomenon solely to climate change or an increase in CO2 concentration in the atmosphere (24). Elevated CO2 concentrations may alter the allergenic potential of peanuts, possibly by increasing the amount of the main peanut allergen, Ara h 1 (25).

Global warming and air pollution are closely linked with inflammation in the upper and lower airways, exacerbating allergic airway diseases (12, 18). The association between exposure to air pollutants early in life and asthma is well-established (19–21). A recent study has suggested that almost 11% of childhood asthma cases are preventable if the recommended precautionary measures are taken (22, 23). It has been proposed that thunderstorms, due to heavy rain, wind gusts, static electricity, and lightning strikes might cause the pollen grains to be ruptured by osmotic shock and form small-sized allergenic particles that can penetrate deep into the lower airways, thereby triggering asthma attacks (3, 25, 26). Recently, there have been multiple acute asthma attacks triggered by thunderstorms during pollen seasons in cities and regions around the world, disrupting the health system (27). Moreover, wildfire smoke induces epithelial barrier dysfunction, causing T cell skewing to T helper (Th) 2 cells, in turn inducing asthma exacerbations (28, 29). Albeit being larger in size, particles in dust storms enhance inflammatory responses and worsen respiratory symptoms (30).

With the effect of climate change, stratospheric ozone is decreasing and the more harmful UVB reaches the surface of the Earth, while increased cloud cover, dust and smoke reduce UV light penetration (31, 32). Ultraviolet (UV) light, cold and dry weather, and floods may aggravate atopic dermatitis (AD). Floods are thought to affect the exacerbations of childhood AD by increasing the levels of indoor molds and contaminated water (33). Moreover, increased concentrations of air pollutants and pollen counts aggravate the severity of AD symptoms (34, 35).

## Epithelial Barrier Hypothesis

To date, several hypotheses have been proposed to explain the complex interplay between the immune system, allergens, environmental triggers, and epigenetics. Damage to the epithelial barriers of the skin, airways and gastrointestinal tract by exposure to harmful substances has been brought to light by the recently proposed “*epithelial barrier hypothesis”* (8). Our journey toward understanding the role of the epithelial barrier in allergic diseases began in 1998 with the first demonstration of keratinocyte apoptosis induced by activated T cells in the pathogenesis of atopic dermatitis (36), asthma and chronic rhinosinusitis (37, 38). In common, these diseases were characterized by the death of highly activated local tissue epithelial cells by apoptosis, which is an important finding in terms of the tissue responses in the physiopathology of these diseases. We demonstrated a chronic epithelial barrier defect, such as spongiosis in the skin, epithelial desquamation in asthma and sinusitis (36–38). Interestingly, highly activated epithelial cells were dying, thereby decreasing the local tissue inflammatory burden. The epithelial barrier was opening and local subepithelial inflammation was draining to the lumens of mucosal surfaces to reduce the epithelial barrier burden. Similarly, dermal inflammation was being drained away from the skin due to spongiotic morphology. Transepidermal water passage, the so-called transepidermal water loss (TEWL), increased in eczema lesions of AD, which contained all the inflammatory cells, cytokines, and chemokines. All these events were decreasing the epithelial inflammatory burden leading to the chronicity of the diseases with a continuous exacerbation and healing process. The opening of the epithelial barrier as an extrusion mechanism also allows for the concomitant entry of allergens, microbes, and toxic substances into deeper tissues (8, 39).

Keep away, wash away, and suppress constitute the main barrier functions of the mucosal tissue, which include multiple immune and tissue cell-related mechanisms (39). First, keep away factors are related to allergen ignorance: increased basement membrane (lamina reticularis) thickness that acts as a physical barrier between allergens and the immune system cells (40), mucosal secretory IgA production against allergens (41), and mucus production in physiological quantities (42). The wash away function involves the clearance of inflammatory cells and cytokines. Mechanisms include opening of the epithelial barriers and clearance of airway tissue inflammatory cells by migration cells, debris and proinflammatory cytokines and chemokines toward the lumen (43), and induction of bronchial epithelial cell apoptosis and their shedding (44). The third function is immune suppression, which includes cells and cytokines that are related to the suppression of inflammation, such as the generation of regulatory dendritic cells (DCs), Treg and Breg cells and their suppressor cytokines (45).

A defective epithelial barrier and the dysregulated interaction between the epithelium, immune cells and microbiota is implicated in the development of multiple allergic conditions (46, 47). The epithelial barrier plays a critical role in maintaining homeostasis through various physiological functions and it acts as the first-line of physical defense and immune response. A leaky epithelium results in dysbiosis of microbial contents which are translocated into interepithelial and subepithelial compartments inducing local tissue inflammation (8). IL-25, IL-33 and thymic stromal lymphopoietin (TSLP) secreted by epithelial cells in response to various stimuli activate local dendritic cells, group 2 innate lymphoid cells and T cells and cause a skew in immune response to Th2 type (48). Th2 cells and their cytokines IL-4 and IL-13, and ILC2s, disrupt epithelial barrier integrity through IL-13 (49, 50).

Both genetic and environmental factors are implicated in the epithelial barriers function. The epidermal barrier in the skin consists of the stratum corneum and TJs. Decreased expression of TJ proteins, such as claudins and occludins, and changes in the composition and dysfunction of lipids (ceramides, free fatty acids and cholesterol) and structural proteins (filaggrin), high skin pH and loss of microbiome diversity may contribute to the epidermal barrier defect observed in patients with atopic dermatitis (51, 52). Loss-of-function mutations in filaggrin also pose risks for the development of food allergy, AR, and asthma. The development of sensitivity to various foods in infants with severe atopic dermatitis, prior to introduction of complementary foods, indicates that this may be associated with an inflamed and impaired epithelial barrier (53).

**Figure 2:**
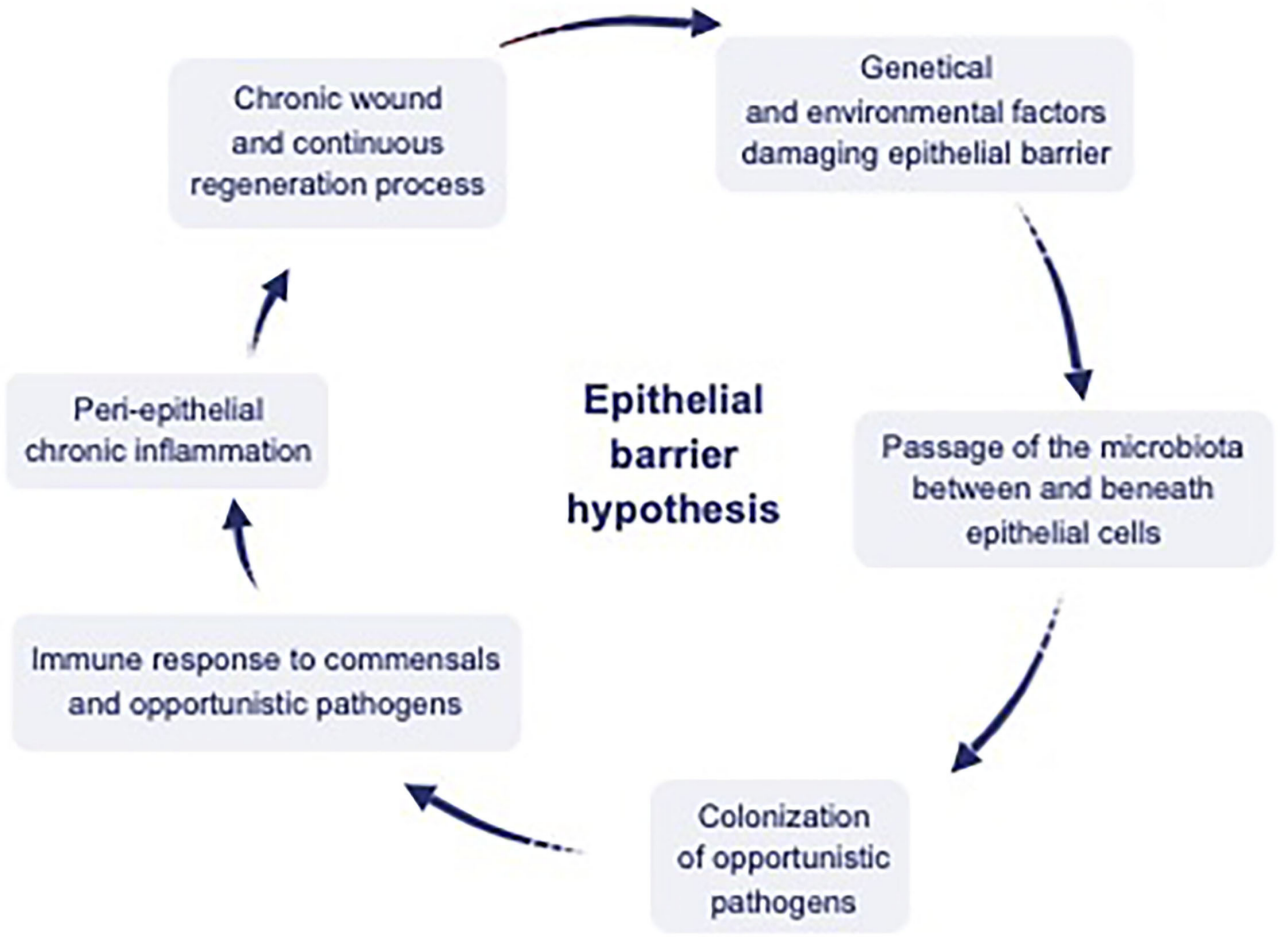
Interaction between environmental factors, epithelium, microbiota, and the immune system. Dysregulation of the epithelial barrier by genetic and environmental factors has been hypothesized to cause a leaky epithelium, which causes dysbiosis of microbial content, including commensals and opportunistic pathogens. Translocation of this content to interepithelial and subepithelial compartments and colonization of the opportunistic pathogens inducing peri-epithelial chronic inflammation. Finally chronic wound and continuous regeneration process ensues [adapted from reference (10)].

Damage of the epithelial barrier integrity by toxic substances initiates a cascade of events that includes changes in the microbial composition and colonization of opportunistic pathogens (Figure 2). A continuous expulsion response develops against microbiota translocating below the epithelium such as the expulsion response to parasites in Loffler's pneumonia (8, 54), which results in microbial dysbiosis and decreased biodiversity. Airway epithelial cells not only form a physical barrier against inhaled particles, but also recruit and activate other effector cells by producing antimicrobial peptides, chemokines, and cytokines to scavenge pathogenic insults (55). Studies have shown that the expression of TJ proteins such as claudin-1, occludin and ZO-1 is significantly reduced in patients with asthma and upper respiratory diseases such as nasal polyps and chronic rhinosinusitis (56, 57). Claudin-18 expression, a TJ protein required for maintaining the integrity of the airway epithelial permeability, was also lower in asthmatics compared to healthy controls (58, 59).

The gastrointestinal epithelial barrier is comprised of desmosomes, adherence junctions and TJs. It represents the largest mucosal surface in contact with foreign antigens and toxins, acting as a selective barrier that absorbs and exchanges nutrients, water, and electrolytes (60). Oral intolerance to foods has been demonstrated to be associated with an impaired intestinal barrier function in various experimental models and clinical trials (61, 62).

## Environmental Substances

There is a long list of toxic substances that directly damage the respiratory, skin, and gut epithelium. Over 200 000 chemicals have been introduced since the 1960s with insufficient data on their toxicity. Our studies have demonstrated that many of these substances damage the epithelial barriers in various tissues, such as those present in cigarette smoke, diesel exhaust, detergents and surfactants, cleaning products, microplastics, nanoparticles, enzymes and emulsifiers in processed food, as well as particulate matter and ozone (63–71). Along with these factors and climate changes, the increasing concentrations and allergenicity of a set of airborne pollens (i.e., oak, ragweed) also needs to be considered (13, 14).

Particulate matter (PM_2.5_ and PM_10_) are one of the most common pollutants, disrupt the epithelial barriers by degrading tight junction proteins (TJ) downregulating occludin and claudin-1, suppressing E-cadherin levels, decreasing transepithelial electrical resistance and increasing paracellular permeability (65, 72). They also disrupt structural proteins such as cytokeratin, filaggrin and cause an increase in lysosomal membrane permeability, lipid peroxidation, and FOXP3 methylation (73). Black carbon produced by incomplete combustion of fuel is an important component of atmospheric PM. Many studies have shown a relationship between exposure to black carbon and the risk of developing asthma (74, 75). In addition, exposure to black carbon has been shown to trigger the development of AR by increasing oxidative stress and inducing the expression of interleukin IL-1β in human nasal epithelial cells (76, 77).

An important traffic-related pollutant, NO_2_, can deeply penetrate and disrupt the epithelial barriers of the upper and lower airways and increase the risk of developing respiratory diseases (74, 75). As another air pollutant, nanoparticles pass the alveolocapillary membrane and can enter the systemic circulation. They directly stimulate epithelial cells, macrophages, and fibroblasts to secrete proinflammatory and profibrotic mediators. Various nanoparticles induce overexpression of immature neurotrophins, destroy phospholipid membranes, endothelial cell junctions and even lysosomal membranes by interacting with lipid-rich structures resulting in epithelial cell death (76). They also disrupt the integrity of the skin and intestinal epithelial barrier with cytotoxic effects by altering cell junctions, inducing proinflammatory cytokines and causing mitochondrial and lysosomal dysfunction (77).

Exposure to ozone can aggravate respiratory diseases by passing through the lower respiratory tract and even to the capillary endothelium, leading to cell stress, desquamation, and cell death by oxidative damage *via* reactive oxygen species (66, 71, 78). In chronic exposure, ozone induces collagen deposition in epithelial and subepithelial areas causing peribronchial fibrosis (78).

There are extensive *in-vivo* and *ex-vivo* studies demonstrating the health hazards of tobacco smoking and e-cigarettes. The toxic chemical substances present induce rapid lipid peroxidation, airway epithelial cell death, impairment of macrophage functions, alter bronchial epithelial cell cytokine secretion patterns and disrupt bronchial epithelial barrier integrity (79, 80).

Direct contact or inhalation of detergents disrupt the epithelial barriers of the skin and respiratory tract, even at very high dilutions or in contact with residues remaining on the laundry after rinsing (7). Surfactants damage the epithelial barrier integrity directly by insulting TJs and related molecules (63, 64) facilitating transepidermal water loss, decreasing stratum corneum hydration and inducing Th2 inflammation (63, 81). Dietary emulsifiers have been demonstrated to alter the microbiota composition in the gut promoting bacteria with an inflammatory potential and facilitates bacterial penetration though the intestinal mucus layer (82).

Microplastics and nanoplastics derived from petroleum can easily infiltrate tissues and interact with cellular structural molecules (83). Microplastics were first solely considered as a pollutant of the oceans. There is increasing evidence indicating high levels of daily human exposure to micro- and nano-plastics through inhalation and ingestion. Once in our respiratory system, nanoplastics can modify the cell lipid membrane structure and secondary structures of proteins, up-regulate pro-inflammatory cytokines, alter the expression of cell cycle-associated proteins, induce inflammatory gene transcription and apoptosis (84).

Overall, particulate air pollutants specially damage the respiratory tract epithelium but also disrupt the skin and gastrointestinal barriers triggering various allergic and inflammatory diseases. Finally, protease activities of airborne allergens such as molds, pollens, cockroaches, and house dust mites have detrimental effects on the TJ molecules in the airway epithelium. In addition, food cysteine proteases also impair the intestinal barrier and increase intestinal permeability (85). Disruption of epithelial barrier integrity facilitates the presentation of allergens and initiates type 2 inflammation (86–88).

## Microbial Dysbiosis

After the development of peri-epithelial inflammation, microbial dysbiosis and decreased biodiversity in the affected tissues are taking place. This has been predominantly attributed to the colonization of opportunistic pathogens in leaky barrier tissues and the mounted immune response against these pathogens. Fundamental changes in modern environments, hygiene and lifestyles reduce microbial biodiversity, leading to more unstable and less resilient microbiota (89). This condition, known as dysbiosis, alters the balance maintained by the gut, skin, and respiratory microbiomes, impairs immune homeostasis and is implicated in many chronic inflammatory diseases, including asthma, allergic diseases such as allergic rhinitis, AD, and food allergy (8, 89–92).

Early exposure to protective commensals contributes to the development of tolerogenic responses (93, 94). The fetal immune system is being influenced and shaped during intrauterine and postnatal periods by maternal infections, microbiota, diet, antibiotics, mode of delivery, breastfeeding, and environmental exposures. These factors may alter the development of lung immunity associated with dysbiosis, contributing to the development of asthma (89, 90, 95). Continuous contact with farm animals increases indoor endotoxin concentrations and this increased exposure promotes an immune regulatory stimulus (96). In addition, microbiota-host interactions have been suggested to play a role in the development of chronic lung diseases. Gut-derived microbiota produce metabolites that reach the lungs (gut-lung axis) and shift the Th2, Treg (Th2-Treg) balance toward Tregs, thereby protecting the host from the development of asthma (89). In a study following 10 years of atopic sensitization and allergic diseases in 180 children aged 7–11 years from Finland and Russia Karelia (both with similar climates, the former is a more Westernized region, the second is a non-Westernized region), Finnish Karelian children were found to be 10 times more likely to develop allergies. Additionally, the bacterial and fungal populations in the nasal mucosa of these participants were less and less diverse than their Russian counterparts. In conclusion, it was stated in this study that exposure to environmental microbiota at an early age may be biologically related to allergic symptoms at younger ages (97). In a different study of young adults in the Karelian region, the number of allergic individuals was found to be lower on the Russian side compared to the Finnish side, which could not be explained by the genotypic difference. This difference was found to be associated with the diversity in the skin and nasal epithelial microbiota of the Russian side and the suppression of the innate immunity by the abundance of acinetobacter on their skin (98, 99)).

Skin microbiome composition and diversity differs between patients with eczema and healthy individuals. There is a decrease in numbers of commensal bacteria in atopic skin, and increased pathogen density in patients with AD is positively correlated with the severity of skin lesions and disease (100). Moreover, intestinal bacterial dysbiosis affects the skin microbiota *via* the gut-skin axis and leads to a systemic imbalance in the Th2-Treg lymphocyte ratio (101).

The gut microbiome is continuously reshaped by external factors such as diet and dysbiosis of the gut microbiome has been associated with the development of food allergy (102). Poor microbiota richness is associated with increased food sensitivity in infants up to 1-year-old (103). Diversity and different taxa were also found to be higher in early life gut microbiota of children with egg allergy (104). This supports the role of specific microbiota in food allergies, opening an avenue for microbiota-targeted therapies.

## Dietary Preferences

There is mounting evidence suggesting that the transition from a traditional diet to a Western diet and increased ratio of n-6/n-3 polyunsaturated fatty acid consumption are associated with the development of allergic diseases. Arachidonic acid (AA), one of the n-6 fatty acid metabolites, aggravates allergic responses through eicosanoids (thromboxane A2, prostaglandin E2 and leukotriene B4) (105). On the other hand, n-3 fatty acids compete with AA and prevent the formation of inflammatory agents through resolvins (especially resolvin E1) (106). Increased consumption of fast food and n-6 fatty acid-derived vegetable oils are the primary dietary insults associated with allergic diseases (107). Although there are limited and some conflicting data, consumption of processed/fast food has been reported to increase the risk of AD (108). A high n-6/n-3 fatty acid ratio induces asthma by causing airway inflammation and bronchoconstriction (109). Contrary to n-6 fatty acids, n-3 fatty acids mainly exert a protective effect on allergic diseases by reducing the inflammatory response, mostly through pro-resolving mediators (110). For example, n-3 fatty acid-derived Protectin D1, generated from docosahexaenoic acid in asthma, ameliorates airway inflammation (111). Administration of n-3 fatty acid supplementation has been found to reduce disease severity in patients with AD (112). In contrast, a different study did not find an association between n-3 fatty acid intake and AD (113). The effect of n-3 fatty acids on food allergy during pregnancy and/or lactation is contradictory. However, n-3 fatty acid supplementation in early pregnancy has been shown to reduce allergic sensitivity to food proteins in the infants (114). In addition, high fecal butyrate levels were associated with a reduced risk of atopic sensitization and the development of asthma and food allergy (115). Moreover, butyrate and propionate have been shown to restore epithelial barrier functions in eosinophilic esophagitis (116).

Oxidative stress is well-recognized to contribute to the pathophysiology of allergic diseases (117). Systemic oxidative stress can aggravate inflammatory responses. Dietary antioxidants and vitamin supplementation have been suggested for reducing the incidence or morbidity of allergic diseases (118). Processed foods contain low levels of antioxidants such as vitamin A, vitamin C and vitamin E and have been suggested to increase the susceptibility to allergic diseases (119). The increase in reactive oxygen species due to insufficient intake of antioxidant vitamins makes individuals susceptible to asthma, AR and AD (120–122). Contrary to these results, there are also studies that don't show any beneficial effects of vitamin supplementation in allergic diseases (123, 124). A diet high in fruits and vegetables, which are good sources of vitamins A, C and E, during lactation and pregnancy and in the infant's diet are protective against the development of food allergy (125). In contrast, high-sugar and fat diets and diet-induced low fecal SCFA levels have been associated with the development of FA (126).

There has been recent controversy on the allergenicity of Genetically Modified (GM) products (127, 128). Some studies have suggested that transgenic crops may have allergic effects, but some do not (129, 130). Long-term studies are warranted to evaluate the risks of consumption of GM foods on the development of food allergies.

In recent years, artificial sweeteners and food additives used to improve taste, color and stability of food products have been increasingly consumed and this may be associated with and increased risk of food allergy, AD, childhood asthma and AR (129–132).

## Conclusion

The external exposome concept is a collective model that not only considers all exposures but also takes into account all of the proven or potential interactions between these environmental factors. Data obtained shows that the rapid increase in the prevalence of allergic diseases and other immune-mediated conditions can be explained by the concept of external exposomes. However, there are gaps in our scientific knowledge on this subject and these need to be answered. For now, it is imperative that we raise awareness of the effects of environmental changes on health to motivate people that will influence governmental policies, for our own well-being and that of future generations.

## Author Contributions

ZC, DM, and CA conceptualized the scope of the article, decided the headings and subheadings and distributed the tasks among all authors. BO and ZC contributed to the part of environmental substances. PC and SA contributed the part of the effects of climate change. MT and IY contributed the part of microbiome. BG and ÜÖ contributed the part of the nutritional factors. CO contributed the part of the epithelial barrier hypothesis. ZC reviewed and combined all parts. ZC, MA, CO, DM, and CA reviewed the article and figures, contributed different sections, and harmonized the final version. All authors contributed to the article and approved the submitted version.

## Conflict of Interest

The authors declare that the research was conducted in the absence of any commercial or financial relationships that could be construed as a potential conflict of interest.

## Publisher's Note

All claims expressed in this article are solely those of the authors and do not necessarily represent those of their affiliated organizations, or those of the publisher, the editors and the reviewers. Any product that may be evaluated in this article, or claim that may be made by its manufacturer, is not guaranteed or endorsed by the publisher.
